# Person-centered care for people with aphasia: tools for shared decision-making

**DOI:** 10.3389/fresc.2023.1236534

**Published:** 2023-10-20

**Authors:** Jacqueline Hinckley, Mark Jayes

**Affiliations:** ^1^ Department of Speech/Language Pathology, Nova Southeastern University, Ft. Lauderdale, FL, United States; ^2^ Department of Health Professions, Manchester Metropolitan University, Manchester, United Kingdom

**Keywords:** aphasia, person-centered, shared decision-making, mental capacity, accessible information, communication disorders, rehabilitation, stroke

## Abstract

Shared decision-making is a fundamental aspect of person-centered care, and can and should be part of many different aspects of the rehabilitation process. Communication disabilities like aphasia, which affects people's ability to use and understand spoken and written language, can make shared decision-making especially challenging to the resources and skills of rehabilitation practitioners. The purpose of this narrative review is to provide a comprehensive description of tools that can support successful shared decision-making with people with aphasia in the rehabilitation environment. These tools and strategies are appropriate for use by physicians, nurses, social workers, physical therapists (also referred to as physiotherapists), occupational therapists, and other service or care providers. The important role of speech-language pathologists as consultants is also described. Case scenarios throughout the paper illustrate the application of recommended tools and strategies along with best practices.

## 1. Introduction

Person-centered care, as carried out during client-provider collaborations across the health care process, is critical to achieving the best possible clinical outcome. The World Health Organization describes person-centered care as an approach that places the perspectives of individuals, families, and communities at the center of how health care services are delivered (1). In this approach, clients are seen as participants in their own health care if they choose, rather than simply recipients of service. For person-centered care to occur, people must be able to receive and understand information about their own condition and express their values, preferences, and decisions to their health care providers.

It has been estimated that 88% of clients in the hospital due to stroke experience one or more communication disabilities, ranging from the language disorder aphasia to the motor speech disorder dysarthria to concomitant cognitive impairments that can affect successful communication (2). In this paper, we will be focusing on aphasia because of the particular way in which it can disrupt person-centered care.

Aphasia is an acquired language disorder due to stroke, brain injury, or other neurological conditions that can limit a person's ability to understand spoken language, speak, read or write. Some evidence suggests that the onset of aphasia with stroke significantly diminishes the likely clinical outcome compared to those with stroke without aphasia; aphasia can increase length of stay, hospital complications, likelihood of mortality, and poorer outcomes in both acute and chronic care (3–6). Although aphasia is often thought of as simply a limited ability to speak, different types of aphasia may have different symptoms in various combinations. One common difficulty in all types of aphasia is word-finding, or having difficulty retrieving the correct word. Most people with aphasia will have some degree of difficulty understanding spoken language, and might have difficulty following directions or conversations. In addition to challenges with speaking and understanding spoken language, people with aphasia may have a range of difficulties with reading and writing. Speech-language pathologists (SLPs) play a critical role in identifying the specific communication challenges of each person, diagnosing the type of aphasia, and making recommendations to the health care team about appropriate communication supports that can help mitigate communication barriers.

The SLP (also known as speech therapist) will characterize the type of aphasia. There are different systems by which aphasia might be categorized (7). For example, aphasia may be described as “fluent” or “nonfluent”, which focuses on speech output. Within those broad categories are more specific syndromes that the SLP may diagnose to signal the constellation of primary symptoms. Another approach, categorizing the aphasia by “receptive” or “expressive”, emphasizes whether understanding spoken language in combination with expressive difficulties is the primary challenge or producing speech is the greater difficulty. The etiology of the aphasia can also potentially cause other communication or cognitive impairments (8), which the SLP can identify and describe how they might affect the person's ability to participate in their own care.

Associated cognitive impairments could include memory, attention, or executive function impairments. Collaboration with other disciplines, such as occupational therapy and neuropsychology, are also important to the success of identification of deficits and management of strategies since some types of aphasia can be mistakenly associated with confusional states or even psychiatric diagnoses (9). The range of aphasic symptoms and possible associated cognitive deficits means that it is not possible to make broad assumptions about the ability of people with aphasia to participate in their own care. In addition, the nature and severity of aphasia or associated impairments is likely to change over time and in response to treatment, necessitating ongoing evaluation about how the person can best participate in their own care and decision-making about care. Like many other things in health care, the process must be personalized to the individual's situation.

The purpose of this narrative review is to provide a comprehensive description of tools that can support successful communication for people with aphasia in the rehabilitation environment and that are appropriate for use by physicians, nurses, social workers, physical therapists (also referred to as physiotherapists), occupational therapists, and other service or care providers. In addition, this may serve as a helpful review for SLPs.

In this paper, we have chosen to refer to people and their roles in conventional ways that are most likely to be recognized by most audiences. We acknowledge that in many contexts “clients” may be referred to as service users, health care users, or health care consumers, among other terms. We will use the term “client” as we find it has the broadest applicability. Formal and informal caregivers may be referred to as carers, co-survivors, family members, partners, or other terms. Finally, we have chosen to refer to health care or social service providers as practitioners.

### 1.1. How is person-centered care affected by aphasia?

Placing the values and preferences of clients at the center of professional practice is a cornerstone of modern ethical care provision that is promoted by international best practice guidance (1). This approach to care has been described in the research and care policy literature using the terms person, patient, or client-centeredness (10). These terms have sometimes been used interchangeably and have been defined in different ways, in the absence of clear theoretical underpinnings. In this paper, we choose to use the term person-centeredness to acknowledge that care is a holistic activity that can occur across a range of settings, not only focused on meeting medical needs in health care environments (11). We also adopt McCormack and McCance's conceptual definition of person-centeredness:

“[A]n approach to practice established through the formation and fostering of healthful relationships between all care providers, service users and others significant to them in their lives. It is underpinned by values of respect for persons, individual right to self-determination, mutual respect and understanding. It is enabled by cultures of empowerment that foster continuous approaches to practice development” [(12), p. 3].

This definition emphasizes the importance of relational aspects of care and links these to quality improvement within care practice. Person-centered care requires practitioners to develop mutually respectful relationships with clients, and other people who are important to them, and to seek to understand these individuals' values and preferences, which, once expressed, guide all aspects of their care and life goals. To achieve this, practitioners need to be able to recognize and acknowledge individuals' unique strengths and support needs, and their inherent competence in understanding their own care needs (11). When the ability to understand or speak is compromised by aphasia or a concomitant cognitive disability, the process of person-centered care can be significantly hampered. Individuals may have difficulty processing technical health information, or they may face challenges in expressing their values, priorities, and preferences for their own care.

Since person-centered care requires a holistic view of the client, an important way in which person-centered care is delivered is through the effective collaboration of practitioners across disciplines and specialties. Collaborative care is defined as meaningful communication between practitioners caring for the same client in a coordinated way that improves the health care outcome; it also includes the important collaboration between client and practitioner. Collaborative care is carried out best when the culture in which it is delivered is built on caring relationships, developing an ownership mentality, providing constructive feedback among team members, applying strengths-based practice, and acting as the first and last lines of defense (13). The SLP is likely to be a cornerstone for other professionals providing care to people with aphasia, because of the insights and tools that the SLP can provide.

The essential elements of a person-centered approach in health care are: (1) an individualized, goal-oriented care plan based on the client's preferences, (2) ongoing review of the person's goals and care plan, (3) care supported by an interprofessional team in which the person is an integral team member, (4) one primary or lead point of contact on the health care team, (5) active coordination among all health care and supportive service providers, (6) continual information sharing and integrated communication, (7) education and training for providers and, when appropriate, the person and those important to the person, and (8) performance measurement and quality improvement using feedback from the person and caregivers (14).

Facilitators that help to achieve person-centered care include communication, team-based care, coordination across transitions, and an organizational culture that provides support and training in person-centered care (14). Indeed, older residents of a long-term care facility identified communication as the key barrier that prevents them from participating in their own care and in decision-making (15). Communication supports, in the forms of tools and strategies but also practitioners' time and expertise, are fundamental to achieving person-centered care.

This central, facilitative role of communication between clients and practitioners becomes even more pertinent when considering practice involving people with aphasia. Aphasia and related communication impairments are known to disrupt usual care and impact the ability of clinicians to provide person-centered care (16). As noted above, people with aphasia and other communication disabilities have specific patterns of communication ability and support needs, and preferred ways of communicating that are unique to each individual. Research suggests some practitioners may engage less successfully in person-centered communication when working with clients who experience communication disabilities (17). Practitioners may not be able to recognize and support the communication needs of clients with aphasia accurately and may choose to seek information about clients with aphasia from their family members and friends rather than communicate with the clients directly (16).

Person-centered care requires practitioners to empower and support individuals to be involved in decision-making about their care, to the extent that they wish to be involved. This means more than seeking informed consent from a person before providing care (18); practitioners need to enable the active involvement of clients in decisions about all aspects of their care, including rehabilitation goal setting, treatment options, and discharge planning. Although people with aphasia have expressed that they want practitioners to involve them in decision-making about their health care and stroke rehabilitation (19, 20), it is important for practitioners to recognize that some clients with aphasia may prefer not to take an active role in decision-making and may defer to practitioners to lead these processes. This could arise for a variety of reasons, including individual differences in what clients understand about the nature and purpose of shared decision-making and their confidence about engaging in it; these issues may be related to, or occur in addition to, individual differences in levels of health literacy and variations in sociocultural attitudes to care (10, 21, 22). However, clients should be offered the opportunity to engage in decision-making.

In person-centered care, decision-making is shared between practitioners and clients, because supporting people to achieve their right to self-determination is considered to be a valuable goal during the care process (21). Shared decision-making represents a collaborative process in which practitioners and clients (and their caregivers) exchange evidence-based information relating to decision options and associated benefits and risks, and to individual values and preferences. This focus on client involvement in clinical decision-making is consistent with most Western healthcare ethical frameworks (23, 24) and established conceptualizations of evidence-based practice (25). Research suggests this approach may help clients to gain enhanced understanding of the relative risks and benefits of decision options and increased satisfaction with the decisions they make and the care plans they form with practitioners. Moreover, shared decision-making has been associated with improved communication and trust between practitioners and clients and greater levels of satisfaction with care (26), increased engagement of clients in the management of their health conditions (27), and enhanced adherence to treatment, superior health outcomes, and reduced health costs (28). Despite this, shared decision-making may still challenge those practitioners who are more familiar with more traditional, paternalistic approaches to clinical decision-making.

During the shared decision-making process, the practitioner's task is to support clients to understand and use this information so that they can consider decision options and their own values and preferences in order to make an informed choice (29, 30). To achieve this, practitioners need to establish what clients understand about decision options and their attitudes towards these. Clearly, shared decision-making depends on effective communication between practitioners and clients (31). As we have already shown, communication processes become more complex when clients have aphasia; communicating in person-centered ways with this group can place greater demands on professionals' resources, including their time (10, 16). This may explain why some practitioners choose to use family members or friends as proxy decision-makers, or make decisions themselves on behalf of a client with aphasia rather than involve the person directly (16, 32). This way of working is unethical when the client might be able to participate given some support, and may not generate accurate information about a client's opinions, wishes and preferences (33). It is important to acknowledge, however, that in some cases the severity of the aphasic or cognitive impairment may so restrict the client's ability to participate that the use of proxies may be necessary.

Practitioners need to be supported to develop their understanding of communication disability and trained to use communication strategies and tools to support individual communication needs. We will describe common strategies and tools in the following sections. Table 1 provides a list of resources that can help practitioners access tools described in this paper. We will also discuss the contribution of SLPs, who have a key role in educating practitioners about person-centered communication and in training them to use specific strategies, approaches and tools to support clients with communication disabilities.

**Table 1:** List of relevant resources, in addition to references, that can help practitioners engage in supported shared decision-making processes with people with aphasia.

Topic area	Brief description	Resource/link
Shared decision-making	Online learning package designed by NICE and University of Keele (UK)	https://www.nice.org.uk/guidance/ng197/resources/shared-decision-making-learning-package-9142488109
Communication tools overview	Overview from the Aphasia Institute in Toronto, ON, CA	https://www.aphasia.ca/communication-tools-communicative-access-sca/
Supported conversation for aphasia ™ training	Training options for practitioners (in-person, online, etc.)	https://www.aphasia.ca/health-care-providers/education-training/
Graphic tools to support conversations with people with aphasia	Graphics, tools, pre-packaged conversation booklets, and other communication supports (some free, others are for purchase)	https://www.aphasia.ca/health-care-providers/resources-and-tools/
Discharge planning	LEAVING checklist from the Australian Aphasia Association	https://www.youtube.com/watch?v=0Z-l2Kv7Aco
Mental capacity assessment	MCAST Support Tool—support to prepare, complete and document inclusive capacity assessments (UK)	https://mmu.estore.flywire.com/products/mental-capacity-assessment-support-toolkit-mcast--support-tool-4801
Mental capacity assessment for people with communication disabilities	Online learning package developed at Manchester Metropolitan University (UK)	https://forms.office.com/e/aAXHB82T6P
Mental capacity assessment for people with communication disabilities	Communication Aid to Capacity Evaluation (CACE)—accessible capacity evaluation process and materials (Canada)	https://aphasia-institute.s3.amazonaws.com/uploads/2021/03/Communication-Aid-to-Capacity-Evaluation-CACE.pdf
Informed consent to participate in research	Consent Support Tool—supports researchers seeking to recruit people with communication disabilities as research participants	https://www.jr-press.co.uk/consent-support-tool.html
Communication support training	Communication Access UK—free online training for individuals and organizations about making communication more accessible	https://communication-access.co.uk/

Practitioners are also likely to benefit from general training in shared decision-making and clients need to be supported to understand the nature of shared decision-making and their role in the process (26). This includes training to use various evidence-based approaches and tools to support shared decision-making (34). Models have been developed to support practitioners to structure discussions about decisions. For example, the “three-talk model” (21, 26) conceptualizes shared decision-making in terms of three steps or stages: “choice talk”, in which the practitioner helps the client to understand that options are available and that they have a right to choose; “option talk”, in which the practitioner provides information about the potential risks, benefits and consequences of each option; and “decision talk”, in which the practitioner supports the client to consider the options, explore their preferences and make an informed decision. Decision support aids are tools that have been developed to support practitioners and clients to explore specific decisions, usually related to medical treatment (34). These aids provide evidence-based, accessible information about the decision, decision options, and associated risks and benefits, and are designed to support clients to consider their own values and preferences when considering this information. However, it should be noted that the majority of this training or these aids have not been designed to meet the specific communication support needs of people with aphasia and therefore may not be accessible to them. We recommend that decision aids are adapted for use with this client group, through the support of SLPs and people with aphasia and their carers.

## 2. How can practitioners communicate with people with aphasia?

In the scenario described in Box 1, the nurse, neurologist, physical therapist and occupational therapist who have seen Terry will benefit from consultation with the SLP, who will be able to specifically assess Terry's ability to understand what is being said and provide appropriate strategies for how the other practitioners can more effectively communicate with Terry. We would argue that it is every practitioner's responsibility to develop knowledge and skills in communicating with people with communication disabilities such as aphasia, but we recognize that such knowledge and skills are often not part of initial practitioner education. SLPs do receive specialist training in this area and have an important role in educating other practitioners to develop the knowledge and skills to use supportive communication techniques effectively during shared decision-making processes. In this section, we will provide an overview of some of those techniques.

### 2.1. What is communication partner training?

Communication Partner Training (CPT) describes several different training approaches in which communication partners of people with aphasia and related impairments learn to use tools and strategies that support comprehension and expression. One specific approach to CPT is Supported Conversation for Aphasia (SCA^TM^) (35), which was originally designed for health care professionals from various disciplines. SCA is accomplished by using a set of communication tools to support comprehension (“Message In”), expression (“Message Out”), and checking to ensure that comprehension has occurred of either message (“Verify”). Table 2 presents a list of sample tools that can be used to help ensure that comprehension, expression, and verification are supported with communication success. The SCA training approach involves first observing use of these tools with specific description and tips, and then actual practice with the tools with people with aphasia. Information about the training, available from the Aphasia Institute in Toronto, Ontario, Canada, is available in Table 1.

**Table 2:** Examples of communication tools and steps that make up Supported Conversation for Adults with Aphasia (SCA^TM^) (35).

Message in	Message out	Verify
• Make sure the individual can hear you and is paying attention • Talk naturally but in short simple sentences • Use gestures while you are talking • Write down the key words that carry the meaning • Use drawing when appropriate • Use resources such as maps, calendars, photos etc.	• Allow time for the individual to get the message out • Ask closed ended questions that use a fixed choice or “yes/no” • Encourage use of gesture— “Can you show me?” • Encourage the individual to write or draw. Give them paper and a marker • Encourage the individual to refer to the key words • Use scales to measure opinion and degrees of feelings	• Explain that you want to make sure that you are understanding correctly • When you are getting a message in check that the person is understanding you • If accuracy is important verify comprehension in two different ways • When the individual is getting the message out verify the message with the use of “yes/no” cards • Expand and verify incomplete messages • Summarize sections of conversation and write down the key words

SCA has been successfully used in many different contexts to train medical students and doctors, nurses, social workers, physical therapists, occupational therapists, and other health care workers. SCA has been implemented as a way to improve communication in health care settings for people with aphasia throughout multiple sites in Denmark (36), and across multinational sites, including Austria, Egypt, Greece, India and Serbia (37).

Part of the success of SCA is perhaps how much value health care workers find in learning these communication tools, because it empowers them to engage the person with aphasia and gather their priorities and preferences. Health care professionals have also found that SCA training enabled them to improve communication with other types of clients, such as clients with post-traumatic amnesia or post-traumatic confusional state due to traumatic brain injury (38).

The critical elements of SCA training for practitioners or students have been observed across multiple studies. Typically, training begins with understanding aphasia and the range of communication strategies that can support successful communication. Importantly, this training must be combined with practice using the strategies with people with aphasia, or trained standardized patients (39–42). Evidence also suggests that both face-to-face training and telepractice training can produce equivalent results (43, 44).

Mental health professionals are just one group of practitioners who do not typically receive sufficient training on how to adapt communication for people with aphasia (45). Mental health professionals acknowledge the need for additional training along with better collaboration with SLPs, so that they can meet the needs of people with aphasia. A large proportion of people with aphasia experience mental health concerns such as depression (approximately 60%) and anxiety (approximately 45%), so communication training for practitioners who can address these concerns is critical (46, 47).

### 2.2. How can practitioners use communication plans to support shared decision-making?

A communication plan is a summary of a person's communication ability and their preferred communication strategies. It should be co-created with the person with aphasia and one or more practitioners, most effectively the SLP. For example, a communication plan might include how a person prefers to receive information, as well as what strategies support effective expression of preferences. If a person has difficulty following lengthy presentations of spoken material, for example, the communication plan might include a preferred strategy, such as writing out key points or pausing after key points to summarize. If a person has difficulty expressing their wishes during a spoken conversation, they may prefer to have time to write key words or point to written choices. Some individuals may prefer to use a digital device, such as an app on a phone or tablet that can support their expression of preferences in the decision-making process. A communication plan clearly summarizes the way in which the person will best receive and understand information, and how they can best express information, but in all cases it must reflect the preference of the person with aphasia on how to accomplish these communication tasks.

In the case of Terry (Box 1), by the end of the first day or two in the hospital, a communication plan could be put in place. After some initial assessment, it might be determined, for example, that Terry can follow spoken language when key words that are being spoken are also written down. It might also be found that Terry can point to words and pictures on a board or digital tablet in order to answer basic questions and verify comprehension. With these communication strategies identified and communicated to all care staff, Terry can fully participate in care, including agreement, consent, or refusal of services.

Box 1 On the first day after hospital admission, the nurse, neurologist, physical therapist and occupational therapist all went in to do an initial consultation. Terry had been admitted to the hospital the night before and diagnosed with a stroke. Sitting up in bed, Terry seemed alert, looking around and following the actions of everyone who entered the room. The neurologist entered and explained about the stroke. Terry replied, “Okay, okay”. The nurse entered and told Terry what medications were being given. Terry said “Okay, okay” and swallowed the pills. When the therapists arrived at different times of the day, Terry said “Okay, okay” and seemed compliant with their requests.

Standing out at the nurses' station, the therapists, doctor and nurse commented that Terry had only been able to say “Okay” and had not been able to repeat any other words. “It seems like Terry understands what we are saying, though”, the physical therapist said. “I’m not so sure about that,” replied the nurse.

Communication plans have repeatedly been shown to be an effective method to improve communication between health care providers and persons with aphasia in hospital, rehabilitation, and long-term residential care settings (48–52). In all cases, success began with an organized staff training of a half to one full day that introduced the importance and process of communication plans. Communication plans were either created prior to the training or during the training. Ongoing support was provided over months or years to help grow the implementation of communication plans into the work environment. Since communication plans have been successful across so many settings and multiple institutions, they are recommended as a realistic and effective way to support communication between practitioners and clients with aphasia and related communication disabilities. A communication plan is a concrete way that shared decision-making about care can occur.

It is important to note that supported practice using the communication plans in the care environment was a critical component to all studies showing success across settings. Practice with communication strategies in an interactive environment with feedback is a key element in the ability of medical students and others to substantially change their communication behaviors, and improve their confidence in interacting with individuals with communication impairments (53–55). Role play and on-the-job coaching are particularly important to the success of any communication partner training (56).

It is also important to note that training a set of communication skills alone does not seem to be sufficient to accomplish the kind of person-centered and supportive communication that proves to be most effective for people with aphasia (57). One approach is to apply the Humanization Values Framework as a way to describe principles and provide examples of humanizing and dehumanizing interactions in the care environment (58). In this model, there are eight dimensions of humanization, which include: (1) embodiment vs. reductionist view of the body, (2) sense-making vs. loss of meaning, (3) sense of place vs. dislocation (4), agency vs. passivity, (5) insiderness vs. objectification, (6) uniqueness vs. homogenization, (7) personal journey vs. loss of personal journey, and (8) togetherness vs. isolation. Regardless of how it is achieved, humanizing interventions, fostering of the therapeutic relationship, and co-constructing engagement are equally important to communication skills training (59–61).

The processes of person-centeredness, collaboration, and shared decision-making underlie many of the fundamental aspects of rehabilitation. In the next sections, we will review how collaboration and shared decision-making improve the effectiveness of consent processes, goal-setting, and transition and discharge planning.

## 3. What needs to be considered when thinking about decision-making capacity for a person with aphasia?

People with aphasia have the legal right to make decisions about all aspects of their lives but may need support to overcome communication barriers associated with their language difficulties to demonstrate their ability to make informed decisions (62). This ability is known as decision-making or mental capacity. Furthermore, people with aphasia may have varying degrees of cognitive difficulty in addition to communication difficulties, which can also create barriers to decision-making. However, it is important to recognize that many people with aphasia can still make informed decisions if they are given individualized support to overcome these barriers (63). Unfortunately, just like in the scenario below involving Mary (Box 2), evidence suggests that some practitioners assume, incorrectly, that clients with aphasia cannot make any decisions for themselves (64). This may be because the presence of communication challenges, such as difficulties understanding what people say, reading, speaking or writing, may mask the true nature of people's decision-making abilities, thereby making it more difficult for practitioners to recognize and acknowledge them (65). This misunderstanding can lead practitioners to under- and over-estimate the decision-making abilities of clients with aphasia (66).

Box 2 Mary is a 79-year-old woman who has aphasia following a previous stroke. Mary's aphasia affects her ability to speak and to understand complex information. She has some word-finding difficulties and dysarthria which make her speech difficult to understand. She also struggles to follow some topics of conversation and complex spoken instructions. Mary is admitted to hospital after a fall. X-rays show that Mary has fractured her hip and requires surgery to repair this. The medical team needs to discuss the surgery with Mary to ensure she understands the nature of the operation, and the potential consequences, including the relative risks and benefits, so that she can give informed consent for them to operate or make a decision not to have surgery. When the doctors speak to Mary, they notice that she appears to have difficulty following their explanation of the surgery. She seems to want to say something about this but is having difficulty expressing herself clearly. The doctors are unsure what to do to help Mary, who becomes increasingly frustrated. Some members of the team think they should ask Mary's son what he thinks about her having the surgery, as they doubt Mary will be able to make this decision for herself.

This has important consequences in terms of person-centered care. As we can see in Mary's case (Box 2), when we under-estimate someone's ability to make decisions, we potentially deny them the right to participate in shared decision-making processes and their right to personal autonomy. This may explain why there is significant evidence that people with aphasia are not given opportunities to engage in decision-making in care settings about many aspects of their daily lives, including what to eat and drink, personal care, medical treatment, and discharge planning; instead others, including practitioners, family members and other advocates, are asked to make such decisions on their behalf (32, 67).

When we overestimate someone's ability to make decisions, we may deny them the support they need to make a decision; this may lead the person to make an uninformed decision. This could mean the person does not fully understand the implications of a decision, which could impact on their ability or willingness to implement the decision; this could have wide ranging negative consequences in a rehabilitation setting, including diminished trust between clients and practitioners and reduced client adherence to treatment (28).

To guard against such misunderstandings, jurisdictions around the world have introduced mental capacity legislative frameworks that are designed to protect individual decision-making rights, for example the Mental Capacity Act in England and Wales (68). Such legislation asserts that all people, regardless of age, diagnosis, or ability, should be presumed to be able to make informed decisions and should be offered individualized support to make decisions if they need this support; this includes support to communicate. In the case scenario above (Box 2), Mary should be offered communication support to help her to make a decision about whether to have the surgery. Therefore, practitioners need to know how to provide communication support to help clients overcome communication barriers during decision-making processes. If Mary had a communication plan in place, this process would be easier.

### 3.1. How can capacity be assessed or evaluated in the presence of aphasia?

In many jurisdictions, the law defines decision-making capacity as time and decision specific and *incapacity* as the inability to make a decision because of an impairment or disturbance of the mind or brain, which could include stroke or aphasia (e.g., Mental Capacity Act (68)). Decision-making capacity legislation asserts that a person must be assumed to be able to make an informed decision unless an assessment of their mental capacity demonstrates otherwise (68). If this process of capacity assessment (sometimes referred to as a capacity evaluation) identifies that an individual lacks capacity to make a specific decision, legal provisions exist to enable others (surrogates) to make decisions on their behalf, in their best interests (69). In that situation, best interests decisions can be made by family members, friends, carers, advocates and practitioners involved in the person's care.

When a capacity assessment identifies that a client lacks capacity to make a decision, practitioners should check if the client has made any decisions in advance about their care, or if another person is legally entitled to do this on their behalf. Different legal frameworks enable individuals to make advanced decisions about aspects of their care, in case they lose the ability to make a decision at a future time. This enables their values, preferences and wishes, indeed their voice, to be included in future decision-making discussions. For example, they might make an advance directive or decision to refuse a specific treatment (68). In some jurisdictions, individuals can also nominate another person, for example a family member, to make decisions on their behalf about their care and other aspects of their lives including financial arrangements. Depending on the legal framework, these people may be referred to as health care proxies (e.g., in the US) or attorneys (in England and Wales). If a person has already lost capacity to make decisions, the courts may appoint someone to do this on their behalf. These people may be referred to as guardians (e.g., in the US) or deputies (e.g., in England and Wales). Practitioners should ensure they understand their responsibilities in relation to the implementation of advance care plans and other aspects of mental capacity legislation that apply in their local jurisdiction.

Practitioners should only complete a mental capacity assessment if they have reason to believe a client may have difficulties making a particular decision, not merely because the individual has a specific health condition (e.g., a stroke) or has communication difficulties (e.g., due to aphasia). A mental capacity assessment tends to involve defined legal tests of the presence of such an impairment or disturbance, usually based on diagnostic or assessment outcome information, and of functional decision-making ability (68). The latter usually takes the form of a conversation-based interview in which the practitioner and client exchange and discuss information about the decision options, as would happen during a shared decision-making process; during a capacity assessment, the practitioner also needs to determine the client's ability to understand, remember and weigh up the information, in order to make and communicate a decision (68).

Thus, in many jurisdictions, legal definitions and tests of decision-making capacity involve the use of linguistic and cognitive abilities (70). People with aphasia or other communication disabilities may not perform as well on these tests unless assessors are skilled at recognizing and supporting their individual communication needs; this could result in clients with aphasia not being able to demonstrate the true nature of their inherent decision-making abilities (69). Unfortunately, evidence suggests that clients with communication disabilities, including those with aphasia, may not always receive the support they are legally entitled to, in order to make decisions (64, 71, 72). Consequently, clients with aphasia are vulnerable during the process of mental capacity assessment and risk being assessed inaccurately (73). This is significant because the outcomes of such assessments may restrict the ability of people with aphasia to maintain their autonomy and independence. Therefore, it is essential that practitioners understand how to complete accurate, legally robust assessments for clients with aphasia; this includes understanding how to provide individualized communication support to enable clients to maximize their participation in decision-making and demonstrate their true decision-making abilities.

### 3.2. How can speech-language pathologists contribute to capacity assessments?

Capacity assessment should be a multidisciplinary responsibility and different members of the team can contribute different areas of expertise (65). For example, occupational therapists and psychologists and mental health professionals can help to identify and implement strategies to support clients' cognitive, mental health and emotional needs. In many jurisdictions, SLPs are experienced in completing capacity assessments for people with aphasia and other communication disabilities and in supporting decision-making more generally. SLPs have significant expertise to bring to the process but may be an underused resource currently (64, 71). To improve this situation, practitioners need to be supported to recognize and understand the role of the SLP in assessments of decision-making capacity for clients with communication disabilities such as aphasia.

SLPs can contribute to capacity assessments directly, by facilitating communication between those responsible for assessing capacity and clients with aphasia during the assessment process or, in certain jurisdictions, by leading capacity assessments themselves (62, 71). SLPs can also contribute indirectly, by educating assessors from other disciplines about communication disability, its relationship with decision-making capacity, and the approaches to supporting communication that can be beneficial during capacity assessments. For example, research suggests that being trained in the Supported Conversation for Aphasia^TM^ [SCA (35)] approach can support practitioners to engage clients with aphasia more successfully in shared decision-making (74) and to complete more inclusive and accurate decision-making capacity assessments (32). SLPs also have a role in training clients with aphasia and their communication partners (e.g., their family members and friends) about these issues, as well as about the legal decision-making and communication support rights of people with communication disabilities.

### 3.3. How can capacity assessment be improved?

Local best practice guidance exists to support practitioners to engage in high quality decision-making capacity assessments, e.g., NICE (75), and research has attempted to identify aspects of practice that appear to facilitate and improve the quality of assessment, e.g., Jayes et al. (76). These sources emphasize the need to ensure individual sensory, communication and cognitive abilities are supported during capacity assessments. Information should be provided and elicited in ways that are tailored to individual communication needs and clients should be supported to communicate during the assessment in whatever ways they find beneficial.

Kagan and colleagues (62) have helpfully provided a comprehensive list of strategies and supports that may be beneficial in supporting people with aphasia to participate in shared decision-making and in decision-making capacity assessments. These strategies and supports are based on the principles of the SCA approach (see Table 2) (35). General strategies include making changes to the communication environment to reduce visual and auditory distractions and giving clients time to process information and respond. Strategies to aid understanding of spoken information include: reducing the amount of information provided and the complexity of language used; emphasizing key words and repeating and rephrasing key information; and supplementing spoken language with visual supports. These include writing, gesture, communication systems such as Talking Mats, and materials such as photographs, symbols, pictograms, and drawings. Written language can be formatted using “aphasia friendly” principles (77) in an attempt to improve its accessibility, as described in Table 3. Many of these strategies can also serve to reduce cognitive load, which is beneficial to people who have aphasia and concomitant cognitive difficulties (63). In addition to the use of these strategies, it is important to consider inviting other people to support the client with aphasia to communicate during the capacity assessment if the client consents to this. These people can include family members, friends and advocates, carers and practitioners.

**Table 3:** Guidelines for aphasia-friendly written material. These guidelines also align with most other recommendations for accessible written material.

Recommendation	Example
Use sentences in simple declarative forms	This is a simple sentence*.* Avoid sentences with multiple parts, such as: *When you come for your appointment, please make sure to wear loose clothing and be on time*.
Shorten all long and complex sentences	Change the sentence above to: *Please come to your appointment on time. Wear loose clothing*.
Change sentences in passive voice to active voice	Change “The aide is called by pressing this button” to: *Press this button*. *The button will call the aide*.
Clarify referents of pronouns	Change “This is a simple sentence” to *This sentence is a simple sentence*.
Substitute more frequent words for infrequent words	Change “Sign the acknowledgment” to: *Sign the form. The form says you received the information*.
Use large print—a minimum of 14-point font	A minimum of 14-point font is recommended. Use sans serif fonts. They are easier to read.
Increase white space (vertical spacing)	Add lines. Separate lines with space.
Useful headings	Add headings that use key words. Make smaller text groups under each heading.
Use lists, tables and bullet points	
Eliminate unnecessary words	
Maintain style and structure throughout document or presentation	
Use high-contrast colors with plain backgrounds	Use high-contrast colors, like black ink on white background. Do not crowd words with complex pictures.
Target a Flesch-Kincaid Reading Grade level of approximately 5–6	Many versions of Word or other word processing applications will calculate readability. You may need to select readability statistics under “Spelling and Grammar” preferences.
Include meaningful images to support the text	Simple pictures are best. The Aphasia Institute provides a free, searchable database of effective pictographs here: https://www.aphasia.ca/participics/.

Sources: PlainLanguage.gov (77), 2011 (78–83).

Evidence-based tools exist to support the process of capacity assessment in different jurisdictions for different types of decisions; for a review, see Lamont et al. (84) and Pennington et al. (85). These tools are designed to improve the validity and reliability of assessments and provide structured approaches to support capacity assessors to give information to clients about available decision options, ask questions to test their specific decision-making abilities, make judgements about their capacity and document this process. Unfortunately, most of these tools were not developed to meet the specific needs of people with communication disabilities such as aphasia.

Positively, in recent years, a small number of evidence-based tools have been developed to use in decision-making capacity assessments for clients with aphasia. The Communication Aid to Capacity Evaluation (CACE) (32) was designed to support social care professionals in Canada to complete capacity evaluations for people with aphasia relating to decisions about residence and care arrangements on discharge from hospital. The CACE includes training in SCA approaches and the use of specific accessible information resources. In the UK, the Mental Capacity Assessment Support Toolkit (MCAST) (86) was designed to enable multidisciplinary professionals to complete legally compliant and holistic capacity assessments. It includes a communication screening tool and photographic resources that practitioners can use to identify how to support clients with communication difficulties, including those with aphasia. Also developed in the UK, the Consent Support Tool (87) was designed to enable researchers to provide information in accessible formats to people with communication disabilities during the informed consent process, to help people make decisions about research participation. Despite these advances, more research is required to develop and evaluate capacity assessment tools to use with people with communication disabilities (75).

## 4. How can shared decision-making be incorporated into goal-setting?

In the scenario described in Box 3, David's transition to outpatient therapy is just one example of when collaborative goal-setting will be critical to the ultimate success of the therapy program. Collaborative goal-setting, such as the Goal Attainment Scaling technique [GAS (88, 89)], is known to improve the overall outcomes of both inpatient and outpatient rehabilitation (90). When collaborative goal-setting techniques are effectively modified to support communication for clients with aphasia, then those with aphasia are able to fully participate and reap the benefits of collaborative goal-setting (91). GAS has been successfully used with people with aphasia to set goals for a treatment program, and also as a personalized and client-reported outcome measure by SLPs (92) and occupational therapists (93).

Despite the many beneficial outcomes of collaborative goal-setting, it can be extremely challenging to use effectively with people with aphasia and other communication disabilities (94). The nature of the communication impairments themselves make collaborative goal setting a challenge. In addition, though, SLPs report feeling a sense of responsibility for leading people with aphasia through the recovery process in a somewhat parental role rather than as equal collaborators, and this may be true for other practitioners as well. In order for a truly collaborative, person-centered process to occur, the practitioner must be able to give up some power and control within the therapeutic relationship and cede some decision-making power to the person with aphasia. This is difficult to do. Although practitioners may often check with clients for approval on goal statements, such approval is a long way from a collaboratively created goal. The implicit power dynamics within the practitioner-client relationship in aphasia seem to be a barrier for the implementation of true person-centered care, particularly as it is instantiated in the goal-setting process. Despite these challenges, the goal-setting process is an important part of the intervention itself and should not be thought of as a purely administrative procedure (95).

Broad recommendations for accessible goal-setting with clients with aphasia have been generated based on a narrative literature review using a systematic search strategy (96). The results showed that aspects of the physical environment, coupled with effective communication techniques, are of utmost importance to collaborative goal-setting. The physical environment should be quiet, with minimized distractions, and offer supports such as paper, pen, pictographs and other tools. A trained and effective communication partner in the context of accessible written materials should use these tools, with easy-to-understand language, and similar support. It was observed that accessibility to information and participation is the responsibility of all practitioners in the environment, not only the SLP. Finally, in support of our previous discussion, it was emphasized that staff training and ongoing support are crucial to the success of implementing accessible goal-setting.

One of the first and perhaps most common types of goal-setting process occurs when an interdisciplinary team meets with the client and family not long after the beginning of care or rehabilitation. These meetings are often called “family meetings”, “team meetings”, or “care planning meetings”, among other terms. In these large group goal-setting meetings, clients with aphasia and their carers generally report feeling listened to and understood (97). Clients with aphasia did report difficulty in being able to pose questions related to their own care in multidisciplinary goal setting meetings. This is an excellent example of an opportunity for a communication plan. If a communication plan had been created in advance with support of the SLP or other practitioner, and distributed in advance to participants and/or reviewed at the start of the meeting, then the chances of full participation, including question-asking, could be substantially improved.

GAS is an evidence-based, effective approach to collaborative goal setting that is used across disciplines. It has long been recommended for use with people with communication disabilities, including aphasia [e.g. (98)], and is one of the more frequently reported aphasia outcome measures by Australian therapists (99). GAS is a process in which the client and practitioner collaborate to select a goal; the client identifies activities or abilities that are the most important, and the practitioner provides expertise relative to task decomposition, sequence of objectives, starting points, and potential treatment approaches. For each goal, the client rates the goal's relative importance and probability of achievement. Baseline ratings are established on a five-point scale, typically −2 to +2, which reflects the expected outcome along with benchmarks that describe below and above expectation. At the end of a predetermined length of treatment, the client and practitioner use the same scale to assess progress.

This process yields clinical goals that are meaningful and relevant to the client, and thus enhance engagement, motivation, and adherence. The goals generated by GAS are often worded in a way that is clear to the client. This facilitates the ability of the client to self-rate importance and achievement. Table 4 provides examples of clinician-directed goal writing compared to collaboratively written goals for the case of David (Box 3).

**Table 4:** Examples of clinician-directed goals and collaborative goals using Goal Attainment Scaling (GAS).

Clinician-directed goals	Collaborative goals (GAS)
1. Client will produce sentences with appropriate verbs in 80% of opportunities	1. Stay in contact with friends and family by writing emails and text messages with more independence
2. Client will improve auditory comprehension of sentences to 85% accuracy	2. Read and understand news articles of interest

Box 3 David was at his desk working as a successful corporate salesman when he had a stroke at age 51 years. As a result of the stroke and aphasia, David was only able to say single words and short phrases. Those words were not always the ones he intended to say, and he had difficulty finding another way to communicate his ideas because his writing was also limited. After a four-week stay in an intensive inpatient rehabilitation program, David was discharged to home with a referral to an outpatient therapy program. During his inpatient rehabilitation stay, SLP goals had included improvement on following multi-step directions and an increase in the frequency of 2–3 word sentences during conversation. During inpatient rehabilitation, he had also achieved his physical therapy goal of being able to move independently and safely around his own home, and his occupational therapy goal of being able to independently dress himself. Now, at the onset of outpatient therapy, David's concerns were focused on his return to work and how he would fill his time productively.

## 5. How can shared decision-making be incorporated into discharge or other transition planning?

Transitions from one treatment or living situation to another, including discharges from treatment, are another pivotal time when shared decision-making is crucial for ensuring that the client's rights, values, and preferences are respected. Too often, transitions and discharges are seen as administrative procedures in which decisions are primarily made by rehabilitation or administrative professionals, with some consultation with the family or other carers. The client who is being discharged or transitioned is often checked with only as a final step, rather than engaging that person at the beginning of the decision-making process. As described in Section 3, typical capacity evaluations that are used to establish whether an individual can consent to a transition plan may not be designed to support the communication needs of individuals with aphasia or related communication impairments, or those with low proficiency in the dominant language (100).

In the case of someone like Emily (Box 4), the transition involves consideration of a move to a different living environment, along with the end of therapies that are being provided in rehabilitation. In this section, we touch on how we can better engage in shared decision-making during both transitions and treatment discharge.

Box 4 Emily is a 78-year old woman who was living alone in an apartment just a short drive away from her daughter, son-in-law, and three grandchildren when she had a stroke. Because of the stroke and aphasia, Emily is able to speak easily but many of the words that she says are not the ones that she intended. She has difficulty understanding what was said to her, and does not always recognize when her own speech was in error. The stroke did not leave Emily with any physical impairment, so she is able to walk and move quite well. The aphasia, however, prevents Emily from effectively using the phone, because she does not always understand what is being said to her, and her speech output contains frequent speech errors embedded in long sentences that are often not understandable. Her daughter took her to her apartment from the rehabilitation facility one time for a short visit. While Emily was overjoyed to see her neighbors, they seemed quite hesitant to interact with Emily.

Emily's stay in the rehabilitation facility is coming to an end in about two weeks. Emily's daughter is adamant that her mom move out of her apartment to an assisted living facility where staff will be available to monitor her safety and provide support. She is also concerned about her mom attempting to drive her car, which is parked at the apartment. Emily is equally adamant about returning to her apartment where she can live independently.

### 5.1. How can practitioners support collaboration in treatment discharge?

Like the difficulties that people with aphasia face in large group care planning meetings, people with aphasia have equally discussed the challenges of asking questions about the discharge process (101, 102). People with aphasia described their confusion about discharge, why therapy was ending, and what would happen afterwards. This suggests that people with aphasia are often not engaged as collaborative partners in a shared decision-making process around discharge. Improved collaborative processes could improve not just discharge but potentially improve the overall outcomes of the therapeutic process itself.

One concept towards discharge from treatment that is often used across disciplines is the idea of “weaning”. Often, therapists have informally been trained to slowly reduce the number or length of treatment visits as the end of the treatment duration approaches. Therapists may feel that they are transferring more responsibility for ongoing improvement to the client with such a schedule, and that they are providing the client the opportunity to take on more home practice. In fact, there is little evidence or formal discussion about this approach to discharge, with a few notable exceptions (103). Hersh (98) describes five categories of strategies by which therapists use weaning: wait-and-see, negotiation, preparation, separation, and replacement.

The Australian Aphasia Association created a discharge checklist that serves as an excellent guide to practitioners on how to engage clients with aphasia in the discharge process and ensure that clients with aphasia understand the process and receive access to appropriate support and resources (104). The checklist is based on published evidence and typical resources available for people with aphasia, and provides a guide based on the acronym LEAVING: Listening, Education, Accessibility to Resources and Support, Validation, Information Technology Supports, Navigating the Health System, and Goals for the Future. The first four steps in the checklist are starting points to ensure that the client's values, preferences and concerns are heard and acknowledged, that the client receives any information or education that is needed, and that the client with aphasia and their carer can clearly state their understanding of the discharge. The final three steps center around ensuring that the client with aphasia and carer have the information needed to access technology supports, such as online groups or health resources, information about how and where to find additional health care services, and what goals the client might have for the future beyond discharge. The checklist is accompanied with an excellent graphic that would support discussion of discharge and could be used by any practitioner or team who is involved in the discharge process. The LEAVING checklist is publicly available online and is listed in Table 1.

### 5.2. How can practitioners better support shared decision-making during transitions to different living situations?

Transitions from one living situation to another seem to be a source of frequent disagreements and tension between clients, caregivers, and practitioners. Often, a root of this tension is the power dynamic between potential decision-makers, and questions about decision-making ability.

In the case of Emily (see Box 4), we must first acknowledge that Emily may have decision-making ability or the ability to contribute importantly to a shared decision-making process. Caregivers and practitioners involved in Emily's case should not assume that Emily cannot participate in determining her next living situation, and her individual autonomy should be respected.

Such a starting point will likely necessitate a productive discussion about Emily's ability to contribute to the decision-making process. Emily is currently in a rehabilitation setting where there is an entire team of rehabilitation professionals who can contribute their respective expertise to a discussion of decision-making capacity. This process may be co-led by the SLP and social worker, for example. Engaging in this discussion will also emphasize to both Emily and her daughter that she has the possibility of participating in the decision-making process. A productive process will require the use of supported communication such as SCA. Tools described in Section 3 might also be appropriate here.

A potential outcome of a discussion of shared decision-making might be the identification of realistic safety concerns, such as the ability to use the phone or other devices to get help. A discussion about this with support communication tools might result in Emily having a better understanding of others' concerns about her welfare, with subsequent better engagement and adherence to important rehabilitation and safety recommendations.

Regardless of the outcome, acknowledging that people with aphasia may have the ability to participate in shared decision-making, and engaging them to the degree possible, is likely to often result in better overall outcomes for everyone involved. Importantly, the client with their preferences, values, and priorities becomes the center of this process.

## 6. Summary and conclusions

The purpose of this narrative review was to provide examples, tools, and strategies that support person-centered care, including shared decision-making, for people with aphasia and related communication disabilities. Person-centered care can challenge practitioners' resources, including time, in all cases, but it may be particularly challenging when a communication disability like aphasia is present.

Shared decision-making is an integral part of providing a person-centered approach in rehabilitation. In every part of the rehabilitation process, from the initial meeting, to explanations of diagnoses, addressing questions and concerns, getting consent for treatment, goal-setting, ensuring the client understands the treatment options and process, discharge from treatment, and transition planning, shared decision-making is fundamental to the provision of best care. These aspects of rehabilitation are most effectively and compassionately done in a truly collaborative process between the client, practitioners, and relevant others such as family members. A number of different tools are available to support practitioners so that they can effectively engage people with aphasia and related communication disabilities in shared decision-making about their own care.

Initially, practitioners are likely to have concerns about the capacity of an individual with a communication disability to participate in decision-making in a meaningful way. Aphasia and other communication disabilities do not necessarily preclude the ability of a person to participate in decision-making; however aphasia may affect decision-making to various degrees. This means that each person should be individually evaluated regarding their ability to participate in decision-making. The ability to participate in shared decision-making may also be an evolving process for some clients, as their medical condition and/or abilities change. Thus, practitioners must continuously consider capacity as they follow a client through time.

For clients who are able to participate in shared decision-making, practitioners must be willing to cede some of the decision-making power, recognizing that allowing a client to choose is likely to result in what is best for the client in their perspective. Ultimately, person-centered care is allowing the client to be the center, and practitioners to truly act in their service.

### 6.1. Implementation considerations

Although several evidence-based approaches are available and are often used by SLPs and others, there are still areas in which more evidence is needed to better inform practice. The relative effectiveness in different settings of the different communication training models, tools and strategies would be helpful, so that those who would like to implement them in their context could do so with the knowledge of what kind of approach is likely to best fit in their system.

Systemic barriers and facilitators play critical roles in whether supported communication and shared decision-making can be integrated in a sustainable way in any given context. Given the differences across settings, including the differences in national and regional policies and procedures, this kind of work is best done within specific contexts. Fortunately, there are known approaches to evaluating and conducting implementation that can be helpful (105, 106). A focus on systems- or policy-level changes not only help to make practices more sustainable, but also more equitable.

### 6.2. Starting points

For those practitioners who would like to expand their practice to better include people with communication disabilities, we would like to recommend the following potential starting points based on the evidence provided in this review. First, participate in supported communication training if you have not done so already. This can be done by collaborating with a SLP in your system, or if one is not available, by accessing resources available in Table 1. The best and most enduring effect will be to create a small group or team who can complete the training together. This initial team can then move on to become “communication champions”, practitioners who watch for opportunities with various clients to support communication through trained techniques. Regular meetings, which can even be short “huddles”, will start to disseminate knowledge about supported communication through the setting. Interest and client successes will help to maintain motivation across practitioners.

A journal club or grand rounds format, which may already exist in many settings, can also be an effective way to reflect on and discuss communication issues that affect shared decision-making and person-centered care. Sources from this review article, or this article itself, could serve as a starting point. Developing a plan with a timeline for how frequently staff will discuss communication issues at meetings will ensure that the team regularly considers these issues.

Consider if there are any policies or procedures within your settings that could be expanded or re-written in a way to bring attention to communication supports for those with communication disabilities. Can aspects of shared decision-making be written into policies that have to do with admission, consent, or discharge planning? Shared decision-making affects all clients, regardless of communication ability or disability, and these considerations should be implemented at all levels.

In order to help future clients and practitioners more readily engage in shared decision-making, students in the health and social services should be trained in supported communication and shared decision-making so that they better provide person-centered care. This training will be most effective if it includes experiential learning and hands-on practice with people with communication disabilities.

Since people with aphasia and other communication disabilities can often participate in decisions that affect them with appropriate support, they should be given that support. It is the obligation of practitioners and educators to provide that support and help to train others to do the same.

## References

[1] World Health Organization. WHO Global strategy on people-centred and integrated health services: Interim report. Geneva: World Health Organization (2015).

[2] O’Halloran R Worrall LE Hickson L. The number of patients with communication related impairments in acute hospital stroke units. Int J Speech Lang Pathol. (2009) 11(6):438–49. 10.3109/17549500902741363 21271921

[3] Boehme AK Martin-Schild S Marshall RS Lazar RM. Effect of aphasia on acute stroke outcomes. Neurology. (2016) 87(22):2348–54. 10.1212/WNL.0000000000003297 27765864 PMC5135027

[4] Lazar RM Boehme AK. Aphasia as a predictor of stroke outcome. Curr Neurol Neurosci Rep. (2017) 17(11):83. 10.1007/s11910-017-0797-z 28929424 PMC13077792

[5] Stransky ML Jensen KM Morris MA. Adults with communication disabilities experience poorer health and healthcare outcomes compared to persons without communication disabilities. J Gen Intern Med. (2018) 33(12):2147–55. 10.1007/s11606-018-4625-1 30143977 PMC6258615

[6] Wu C Qin Y Lin Z Yi X Wei X Ruan Y Prevalence and impact of aphasia among patients admitted with acute ischemic stroke. J Stroke Cerebrovasc Dis. (2020) 29(5):104764. 10.1016/j.jstrokecerebrovasdis.2020.104764 32173230

[7] Sheppard SM Sebastian R. Diagnosing and managing post-stroke aphasia. Expert Rev Neurother. (2021) 21(2):221–34. 10.1080/14737175.2020.1855976 33231117 PMC7880889

[8] Mitchell C Gittins M Tyson S Vail A Conroy P Paley L Prevalence of aphasia and dysarthria among inpatient stroke survivors: describing the population, therapy provision and outcomes on discharge. Aphasiology. (2021) 35(7):950–60. 10.1080/02687038.2020.1759772

[9] Pallickal M Hema N. Discourse in wernicke’s aphasia. Aphasiology. (2020) 34(9):1138–63. 10.1080/02687038.2020.1739616

[10] Forsgren E Åke S Saldert C. Person-centred care in speech-language therapy research and practice for adults: a scoping review. Int J Lang Commun Disord. (2022) 57(2):381–402. 10.1111/1460-6984.12690 34997806

[11] Håkansson Eklund J Holmström IK Kumlin T Kaminsky E Skoglund K Höglander J Same same or different? A review of reviews of person-centered and patient-centered care. Patient Educ Couns. (2019) 102(1): 3–11. 10.1016/j.pec.2018.08.029 30201221

[12] McCormack B McCance T. Person-centred practice in nursing and health care, theory and practice. Oxford: Blackwells (2017).

[13] Wei H Corbett RW Ray J Wei TL. A culture of caring: the essence of healthcare interprofessional collaboration. J Interprof Care. (2020) 34(3):324–31. 10.1080/13561820.2019.1641476 31390903

[14] American Geriatric Society Expert Panel on Person-Centered Care. Person-centered care: a definition and essential elements. J Am Geriatr Soc. (2016) 64(1):15–8. 10.1111/jgs.13866 26626262

[15] Bennett M von Treuer K McCabe MP Beattie E Karantzas G Mellor D Resident perceptions of opportunity for communication and contribution to care planning in residential aged care. Int J Older People Nurs. (2020) 15(1):e12276. 10.1111/opn.12276 31578823

[16] Carragher M Steel G O’Halloran R Torabi T Johnson H Taylor NF Aphasia disrupts usual care: the stroke team’s perceptions of delivering healthcare to patients with aphasia. Disabil Rehabil. (2021) 43(21):3003–14. 10.1080/09638288.2020.1722264 32045533

[17] Skinder-Meredith A Bye L Bulthuis K Schueller A. Patient-centered communication survey of nursing homes and rehabilitation centers. Care Manag J. (2007) 8(1):8–15. 10.1891/152109807780494078 17491445

[18] King JS Moulton BW. Rethinking informed consent: the case for shared medical decision-making. Am J Law Med. (2006) 32(4):429–501. 10.1177/009885880603200401 17240730

[19] Berg K Askim T Balandin S Armstrong E Rise MB. Experiences of participation in goal setting for people with stroke-induced aphasia in Norway. A qualitative study. Disabil Rehabil. (2017) 39(11):1122–30. 10.1080/09638288.2016.1185167 27293106

[20] Hersh D Worrall L Howe T Sherratt S Davidson B. SMARTER Goal setting in aphasia rehabilitation. Aphasiology. (2012) 26(2):220–33. 10.1080/02687038.2011.640392

[21] Elwyn G Frosch D Thomson R Joseph-Williams N Lloyd A Kinnersley P Shared decision making: a model for clinical practice. J Gen Intern Med. (2012) 27(10):1361–7. 10.1007/s11606-012-2077-6 22618581 PMC3445676

[22] Isaksen J. “‘well, you are the one who decides’ attempting shared decision making at the end of aphasia therapy.” Top Lang Disord. (2018) 38:2. 10.1097/TLD.0000000000000150

[23] Beauchamp TL Childress JF. Principles of biomedical ethics. Oxford: Oxford University Press (2008).

[24] Seedhouse D. Ethics: The heart of health care. Chichester, UK: Wiley-Blackwell (2009).

[25] Sackett DL Straus SE Richardson WS Rosenberg W Haynes RB. Evidence based medicine: How to practise and teach EBM. London: Churchill Livingstone (2000).

[26] National Institute for Health and Care Excellence (NICE). Shared decision making. NICE Guideline NG197 (2021). Available at: https://www.nice.org.uk/guidance/ng197/resources/shared-decision-making-pdf-66142087186885 34339147

[27] Hoffmann TC Légaré F Simmons MB McNamara K McCaffery K Trevena LJ Shared decision making: what do clinicians need to know and why should they bother? Med J Aust. (2014) 201(1):35–9. 10.5694/mja14.00002 24999896

[28] Bunn F Goodman C Russell B Wilson P Manthorpe J Rait G Supporting shared decision making for older people with multiple health and social care needs: a realist synthesis. BMC Geriatr. (2018) 18:165. 10.1186/s12877-018-0853-9 30021527 PMC6052575

[29] Charamis K Parsons C Leonard C Domecq MC Smith FM Mayhew KJ Shared decision making for persons with aphasia: a scoping review. Aphasiology. (2023) 37(5):802–12. 10.1080/02687038.2022.2039588

[30] Elwyn G Laitner S Coulter A Walker E Watson P Thomson R. Implementing shared decision making in the NHS. Br Med J. (2010) 341:c5146. 10.1136/bmj.c5146 20947577

[31] Stewart M Brown JB Donner A McWhinney IR Oates J Weston WW The impact of patient-centered care on outcomes. J Fam Pract. (2000) 49(9):796–804. 11032203

[32] Carling-Rowland A Black S McDonald L Kagan A. Increasing access to fair capacity evaluation for discharge decision-making for people with aphasia: a randomised controlled trial. Aphasiology. (2014) 28(6):750–65. 10.1080/02687038.2014.895975

[33] Haley KL Womack J Helm-Estabrooks N Lovette B Goff R. Supporting autonomy for people with aphasia: use of the life interests and values (LIV) cards. Top Stroke Rehabil. (2013) 20(1):22–35. 10.1310/tsr2001-22 23340068

[34] Stacey D Legare F Lewis K Barry MJ Bennett CL Eden KB Decision aids for people facing health treatment or screening decisions. Cochrane Database Syst Rev. (2017) 4:CD001431. 10.1002/14651858.CD001431.pub5 28402085 PMC6478132

[35] Kagan A. Supported conversation for adults with aphasia: methods and resources for training conversation partners. Aphasiology. (1998) 12(9):816–30. 10.1080/02687039808249575

[36] Isaksen J Jensen LR. The (S)CAse of Denmark: multisite implementation of supported conversation for adults with aphasia (SCA™). Aphasiology. (2018) 32(sup1):101–2. 10.1080/02687038.2018.1487015

[37] Isaksen J Beeke S Pais A Efstratiadou E-A Pauranik A Revkin SK Communication partner training for healthcare workers engaging with people with aphasia: enacting sustainable development goal 17 in Austria, Egypt, Greece, India and Serbia. Int J Speech Lang Pathol. (2023) 25(1):172–7. 10.1080/17549507.2022.2145355 36927168

[38] Nielsen AI Jensen LR Power E. Meeting the confused patient with confidence: perceived benefits of communication partner training in subacute TBI. Brain Inj. (2023) 37(3):208–21. 10.1080/02699052.2022.2158224 36571429

[39] Baylor C Burns M McDonough K Mach H Yorkston K. Teaching medical students skills for effective communication with patients who have communication disorders. Am J Speech Lang Pathol. (2019) 28(1):155–64. 10.1044/2018_AJSLP-18-0130 31072161 PMC6503863

[40] Legg C Young L Bryer A. Training sixth-year medical students in obtaining case-history information from adults with aphasia. Aphasiology. (2005) 19(6):559–75. 10.1080/02687030544000029

[41] Shrubsole K Lin T-J Burton C Scott J Finch E. Delivering an iterative communication partner training programme to multidisciplinary healthcare professionals: a pilot implementation study and process evaluation. Int J Lang Commun Disord. (2021) 56(3):620–36. 10.1111/1460-6984.12618 33818902

[42] van Rijssen M Veldkamp M Meilof L van Ewijk L. Feasibility of a communication program: improving communication between nurses and persons with aphasia in a peripheral hospital. Aphasiology. (2019) 33(11):1393–409. 10.1080/02687038.2018.1546823

[43] Cameron A McPhail S Hudson K Fleming J Lethlean J Finch E. Telepractice communication partner training for health professionals: a randomised trial. J Commun Disord. (2019) 81:105914. 10.1016/j.jcomdis.2019.105914 31229734

[44] Power E Falkenberg K Barnes S Elbourn E Attard M Togher L. A pilot randomized controlled trial comparing online versus face-to-face delivery of an aphasia communication partner training program for student healthcare professionals. Int J Lang Commun Disord. (2020) 55(6):852–66. 10.1111/1460-6984.12556 32654395

[45] Strong KA Randolph J. How do you do talk therapy with someone who can’t talk? Perspectives from mental health providers on delivering services to individuals with aphasia. Am J Speech Lang Pathol. (2021) 30(6):2681–92. 10.1044/2021_AJSLP-21-00040 34674537

[46] Laures-Gore JS Dotson VM Belagaje S. Depression in poststroke aphasia. Am J Speech Lang Pathol. (2020) 29(4):1798–810. 10.1044/2020_AJSLP-20-00040 33181048

[47] Morris R Eccles A Ryan B Kneebone II. Prevalence of anxiety in people with aphasia after stroke. Aphasiology. (2017) 31(12):1410–5. 10.1080/02687038.2017.1304633

[48] Forsgren E Saldert C. Implementation of communication routines facilitating person-centred care in long-term residential care: a pilot study. Health Expect. (2022) 25(6):2982–91. 10.1111/hex.13606 36148650 PMC9700177

[49] Généreux S Julien M Larfeuil JC Lavoie V Soucy O Le Dorze G. Using communication plans to facilitate interactions with communication-impaired persons residing in long-term care institutions. Aphasiology. (2004) 18(12): 1161–75. 10.1080/02687030444000507

[50] McGilton K Sorin-Peters R Sidani S Rochon E Boscart V Fox M. Focus on communication: increasing the opportunity for successful staff-patient interactions. Int J Older People Nurs. (2011) 6(1):13–24. 10.1111/j.1748-3743.2010.00210.x 21303460

[51] McGilton KS Sorin-Peters R Rochon E Boscart V Fox M Chu CH The effects of an interprofessional patient-centered communication intervention for patients with communication disorders. Appl Nurs Res. (2018) 39:189–94. 10.1016/j.apnr.2017.11.017 29422157

[52] Sorin-Peters R McGilton KS Rochon E. The development and evaluation of a training programme for nurses working with persons with communication disorders in a complex continuing care facility. Aphasiology. (2010) 24(12):1511–36. 10.1080/02687038.2010.494829

[53] Forsgren E Hartelius L Saldert C. Improving medical students’ knowledge and skill in communicating with people with acquired communication disorders. Int J Speech Lang Pathol. (2017) 19(6):541–50. 10.1080/17549507.2016.1216602 27576788

[54] Horton S Lane K Shiggins C. Supporting communication for people with aphasia in stroke rehabilitation: transfer of training in a multidisciplinary stroke team. Aphasiology. (2016) 30(5):629–56. 10.1080/02687038.2014.1000819

[55] Jensen LR Løvholt AP Sørensen IR Blüdnikow AM Iversen HK Hougaard A Implementation of supported conversation for communication between nursing staff and in-hospital patients with aphasia. Aphasiology. (2015) 29(1):57–80. 10.1080/02687038.2014.955708

[56] van Rijssen M Ketelaar M Vandenborre D Oostveen J Veldkamp M van Ewijk L Evaluating communication partner training in healthcare centres: understanding the mechanisms of behaviour change. Int J Lang Commun Disord. (2021) 56(6):1190–203. 10.1111/1460-6984.12659 34370352

[57] Rowland A McDonald L. Evaluation of social work communication skills to allow people with aphasia to be part of the decision making process in healthcare. Soc Work Educ. (2009) 28(2):128–44. 10.1080/02615470802029965

[58] Pound C Jensen LR. Humanising communication between nursing staff and patients with aphasia: potential contributions of the humanisation values framework. Aphasiology. (2018) 32(10):1225–49. 10.1080/02687038.2018.1494817

[59] Bright FA Kayes NM Cummins C Worrall LM McPherson KM. Co-constructing engagement in stroke rehabilitation: a qualitative study exploring how practitioner engagement can influence patient engagement. Clin Rehabil. (2017) 31(10):1396–405. 10.1177/0269215517694678 28653548 PMC5613802

[60] Gordon C Ellis-Hill C Dewar B Watkins C. Knowing-in-action that centres humanising relationships on stroke units: an appreciative action research study. Brain Impair. (2022) 23(1):60–75. 10.1017/BrImp.2021.34

[61] Kayes NM Cummins C McPherson KM Worrall L Bright FAS. Developing connections for engagement in stroke rehabilitation. Brain Impair. (2022) 23(1):42–59. 10.1017/BrImp.2021.27

[62] Kagan A Shumway E MacDonald S. Assumptions about decision-making capacity and aphasia: ethical implications and impact. Semin Speech Lang. (2020) 41(03):221–31. 10.1055/s-0040-1712115 32585706

[63] Kim ES Suleman S Hopper T. Decision making by people with aphasia: a comparison of linguistic and nonlinguistic measures. J Speech Lang Hear Res. (2020) 63(6):1845–60. 10.1044/2020_JSLHR-19-00182 32464071

[64] McCormick M Bose A Marinis T. Decision-making capacity in aphasia: sLT’s contribution in England. Aphasiology. (2017) 31(11):1344–58. 10.1080/02687038.2017.1355441

[65] Zuscak SJ Peisah C Ferguson A. A collaborative approach to supporting communication in the assessment of decision-making capacity. Disabil Rehabil. (2016) 38(11):1107–111. 10.3109/09638288.2015.1092176 26458145

[66] Kagan A Kimelman MDZ. Informed consent in aphasia research: myth or reality? Clin Aphasiol. (1995) 23:65–75. 10.1080/02687030701521786

[67] van Rijssen M Isaksen J Vandenborre D Veldkamp M Bryon E Remijn L Ways to improve communication and support in healthcare centres according to people with aphasia and their relatives: a Dutch perspective. Aphasiology. (2023) 37(1):69–82. 10.1080/02687038.2021.1988505

[68] Mental Capacity Act. England, Wales, London: Office of Public Sector Information (2005).

[69] Wagner LCB. Ethical framework of supporting medical decision making for persons with aphasia. Perspect ASHA Special Interest Groups. (2018) 3(2):80–7. 10.1044/2018_PERS-SIG2-2018-0014

[70] Suleman S Hopper T. Decision-making capacity and aphasia: speech-language pathologists’ perspectives. Aphasiology. (2016) 30(4):381–95. 10.1080/02687038.2015.1065468

[71] Borrett S Gould LJ. Mental capacity assessment with people with aphasia: understanding the role of the speech and language therapist. Aphasiology. (2021) 35(11):1463–81. 10.1080/02687038.2020.1819954

[72] Jayes M Austin L Brown LJE. Supported decision-making and mental capacity assessment in care homes: a qualitative study. Health Soc Care Community. (2022) 30(4):E1061–9. 10.1111/hsc.13512 34250675

[73] Ferguson A Duffield G Worrall L. Legal decision-making by people with aphasia: critical incidents for speech pathologists. Int J Lang Commun Disord. (2010) 45(2):244–58. 10.3109/13682820902936714 22748035

[74] Simmons-Mackie NN. Communicative access and decision making for people with aphasia: implementing sustainable healthcare systems change. Aphasiology. (2007) 21(1):39–66. 10.1080/02687030600798287

[75] National Institute for Health and Care Excellence (NICE). Decision-making and mental capacity. NICE guideline NG108 (2018). Available at: https://www.nice.org.uk/guidance/ng108/resources/decisionmaking-and-mental-capacity-pdf-66141544670917

[76] Jayes M Palmer R Enderby P Sutton A. How do health and social care professionals in England and Wales assess mental capacity? A literature review. Disabil Rehabil. (2020) 42(19):2797–808. 10.1080/09638288.2019.1572793 30739505

[77] United States Government. *Plain language*. (2023). Available at: https://www.plainlanguage.gov/

[78] Rose TA Worrall LE McKenna KT. The effectiveness of aphasia-friendly principles for printed health education materials for people with aphasia following stroke. Aphasiology. (2003) 17(10):947–63. 10.1080/02687030344000319

[79] Aleligay A Worrall LE Rose TA. Readability of written health information provided to people with aphasia. Aphasiology. (2008) 22(4):383–407. 10.1080/02687030701415872

[80] Brennan AD Worrall LE McKenna KT. The rellationship between specific features of aphasia-friendly written material and comprehension of written material for people with aphasia: an exploratory study. Aphasiology. (2005) 19(8):693–711. 10.1080/02687030444000958

[81] Herbert R Haw C Brown C Gregory E Brumfitt S, Stroke Association Accessible Information Guidelines. (2012). Available at: http://www.stroke.org.uk/sites/default/files/Accessible%20Information%20Guidelines.pdf.pdf

[82] National Health Service (NHS) England. *Accessible information standard*. (2017). Available at: https://www.england.nhs.uk/about/equality/equality-hub/patient-equalities-programme/equality-frameworks-and-information-standards/accessibleinfo/

[83] Saylor A Wallace SE Brown ED Schreiber J. Aphasia-friendly medication instructions: effects on comprehension in persons with and without aphasia. Aphasiology. (2022) 36(3):251–67. 10.1080/02687038.2021.1873907

[84] Lamont S Jeon YH Chiarella M. Assessing patient capacity to consent to treatment: an integrative review of instruments and tools. J Clin Nurs. (2013) 22(17–18):2387–403. 10.1111/jocn.12215 23650907

[85] Pennington C Davey K ter Meulen R Coulthard E Kehoe PG. Tools for testing decision-making capacity in dementia. Age Ageing. (2018) 47(6):778–84. 10.1093/ageing/afy096 30010696

[86] Jayes M Palmer R Enderby P. Evaluation of the MCAST, a multidisciplinary toolkit to improve mental capacity assessment. Disabil Rehabil. (2022) 44(2):323–30. 10.1080/09638288.2020.1765030 32449375

[87] Palmer R Jayes M. The consent support tool. Albury, UK: J & R Press (2020).

[88] Turner-Stokes L. Goal attainment scaling (GAS) in rehabilitation: a practical guide. Clin Rehabil. (2009) 23(4):362–70. 10.1177/0269215508101742 19179355

[89] Turner-Stokes L Williams H Johnson J. Goal attainment scaling: does it provide added value as a person-centred measure for evaluation of outcome in neurorehabilitation following acquired brain injury? J Rehabil Med. (2009) 41(7):528–35. 10.2340/16501977-0383 19543663

[90] Grant M Ponsford J. Goal attainment scaling in brain injury rehabilitation: strengths, limitations and recommendations for future applications. Neuropsychol Rehabil. (2014) 24(5):661–77. 10.1080/09602011.2014.901228 24787703

[91] Elston A Barnden R Hersh D Godecke E Cadilhac DA Lannin NA Developing person-centred goal setting resources with and for people with aphasia: a multi-phase qualitative study. Aphasiology. (2022) 36(7):761–80. 10.1080/02687038.2021.1907294

[92] Doogan C Al Balushi R Gooding B Crinion J Leff A. What do people with aphasia want from the queen square intensive comprehensive aphasia programme and do they achieve it? A quantitative and qualitative analysis of their short, medium, long-term and economic goals. Aphasiology. (2023) 37(10):1661–78. 10.1080/02687038.2022.2118518

[93] Escher AA Amlani AM Viani AM Berger S. Occupational therapy in an intensive comprehensive aphasia program: performance and satisfaction outcomes. Am J Occup Ther. (2018) 72(3):7203205110p1–205110p7. 10.5014/ajot.2018.026187 29689180

[94] Berg K Askim T Rise MB. What do speech-language pathologists describe as most important when trying to achieve client participation during aphasia rehabilitation? A qualitative focus group interview study. Int J Speech Lang Pathol. (2019) 21(5):493–503. 10.1080/17549507.2017.1413134 29252012

[95] Evans JJ. Goal setting during rehabilitation early and late after acquired brain injury. Curr Opin Neurol. (2012) 25(6):651–5. 10.1097/WCO.0b013e3283598f75 23007008

[96] Brown SE Brady MC Worrall L Scobbie L. A narrative review of communication accessibility for people with aphasia and implications for multi-disciplinary goal setting after stroke. Aphasiology. (2021) 35(1):1–32. 10.1080/02687038.2020.175926935002009

[97] Söderhielm K Eriksson K Möller M. Communicative participation in goal-setting meetings for patients with aphasia after stroke. A study using patients’ and healthcare professionals’ self-ratings. Int J Lang Commun Disord. (2023) 58(2):342–56. 10.1111/1460-6984.12791 36218168

[98] Worrall L Frattali C, Eds. Neurogenic communication disorders: A functional approach. New York: Thieme. (2000). p. 59–65.

[99] Arnold H Wallace SJ Ryan B Finch E Shrubsole K. Current practice and barriers and facilitators to outcome measurement in aphasia rehabilitation: a cross-sectional study using the theoretical domains framework. Aphasiology. (2020) 34(1):47–69. 10.1080/02687038.2019.1678090

[100] Carling-Rowland A Wahl J. The evaluation of capacity to make admission decisions: is it a fair process for idividuals with communication barriers? Med Law Int. (2010) 10(3):171–90. 10.1177/096853321001000301

[101] Hersh D. Experiences of ending aphasia therapy. Int J Lang Commun Disord. (2001) 36(S1):80–5. 10.3109/13682820109177863 11340849

[102] Hersh D. How do people with aphasia view their discharge from therapy? Aphasiology. (2009) 23(3):331–50. 10.1080/02687030701764220

[103] Hersh D. “Weaning” clients from aphasia therapy: speech pathologists’ strategies for discharge. Aphasiology. (2003) 17(11):1007–29. 10.1080/02687030344000364

[104] Australian Aphasia Association. *AAA discharge planning LEAVING checklist for people with aphasia*. (2020). Available at: https://aphasia.org.au/wp-content/uploads/2020/04/Discharge-Planning-LEAVING-checklist.pdf

[105] Abbott KM Douglas N Van Haitsma K. A rising tide lifts all boats: equitable nursing home policy through implementation science. Public Policy Aging Rep. (2022) 32(1):6–12. 10.1093/ppar/prab030

[106] Grover S Fitzpatrick A Azim FT Ariza-Vega P Bellwood P Burns J Defining and implementing patient-centered care: an umbrella review. Patient Educ Couns. (2022) 105(7):1679–88. 10.1016/j.pec.2021.11.004 34848112

